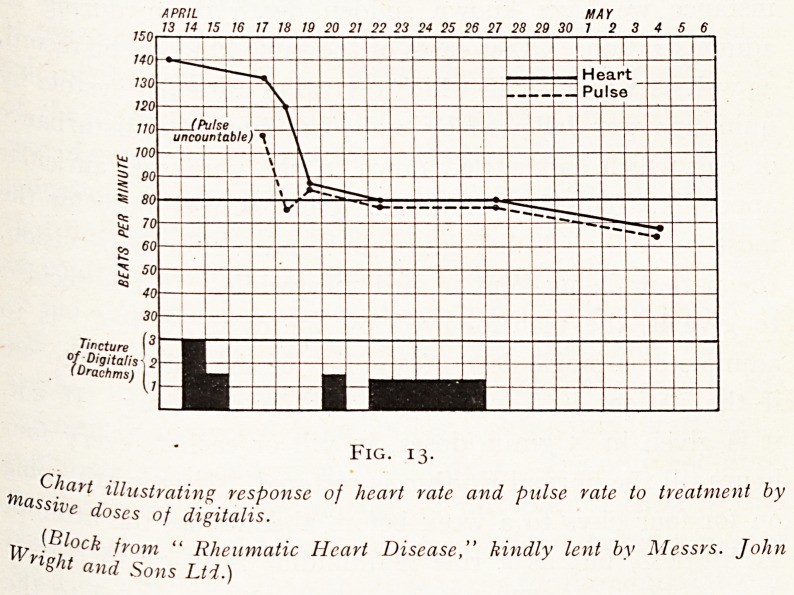# Auricular Failure
1We have to express our sincere thanks to the Colston Research Society for helping us to defray the expenses of the investigations alluded to in this paper, as well as those of the illustrations; to Dr. Geoffrey Hadfield for furnishing us with the photomicrographs; and to Messrs. John Wright and Sons for the loan of blocks from a book on Rheumatic Heart Disease which they are just bringing out.


**Published:** 1924-10

**Authors:** Carey F. Coombs, C. E. K. Herapath

**Affiliations:** Physician (with charge of Out-patients), Bristol General Hospital; Assistant Physician, Bristol Royal Infirmary


### AURICULAR FAILURE.[1]

BY

CAREY F. COOMBS, M.D., F.R.C.P. Lond.,

*Physician (with charge of Out-patients), Bristol General Hospital.*

AND

C. E. K. HERAPATH, M.C., M.D. Lond.

*Assistant Physician, Bristol Royal Infirmary.*

---

THE effect of auricular failure on the heart has to be considered from two standpoints; first, the effect of the loss of the auricular contraction, and second, the effect of the abnormal rhythm of auricular failure on the ventricular contraction.

### ANATOMY AND PHYSIOLOGY.

The function of the auricles is to act as reservoirs for the ventricles. The blood which collects in them during the ventricular systole passes into the ventricles at the commencement of diastole; and as diastole goes on, first the ventricles fill and then the auricles. The auricular contraction then distends the already filled ventricles, making certain that a full amount of blood shall be pumped into the aorta and pulmonary arteries every time that the ventricles contract. Loss of the auricular contraction will, on that account, lead to an insufficient filling of the ventricles. The effect of abnormal rhythms of the auricles on the ventricular contraction will be considered later.

[1] We have to express our sincere thanks to the Colston Research Society for helping us to defray the expenses of the investigations alluded to in this paper, as well as those of the illustrations; to Dr. Geoffrey Hadfield for furnishing us with the photomicrographs; and to Messrs. John Wright and Sons for the loan of blocks from a book on *Rheumatic Heart Disease* which they are just bringing out.

FIG. 1.

*Photograph of heart, showing position of sinu-auricular node.*

FIG. 2.

*Photograph of heart with right auricle and ventricle opened to show position of auriculo-ventricular node and bundle. The rectangular outline is that of the block cut out to provide serial sections of these structures. The block is cut transversely for this purpose.*

FIGS. 3 and 4.

*Sinu-auricular node.*

*The upper (low-power) photomicrograph shows the general structure of the node. The darker area is that of the general auricular muscle ; the rest is that of the node, with its central vessels. The lower (high-power) one shows a nerve trunk N sending its fibres into the substance of the node, which is mainly composed of bundles of fine muscle fibres.*

The contraction of the heart is started at the upper part of the right auricle by a collection of cells known as the sinu-auricular node (Fig. 1). This node is situated in the wall of the right auricle, where the superior vena cava joins the right auricle and the base of the right auricular appendix. It is an elongated structure lying at right angles to the length of the superior vena cava, and is continued for a small distance down the sulcus terminalis.

It is composed of dense fibrous tissue in which are embedded small fine muscle fibres (Fig. 3). A large number of nerve fibres lead into it (Fig. 4). This collection of cells has the property of originating a stimulus for the contraction of the heart muscle.

Much controversy has taken place as to the path by which this stimulus is carried on to the auriculo-ventricular node. Work has been published stating that there is a direct path from node to node, and this is said to have been proved by electrical means. Three years ago we set out to look for this path by means of serial sections of hearts. Six hearts were cut and examined, and the results of this search proved to us that there was no direct path, but that the stimulus passes to the A–V node by means of the ordinary auricular muscle, the contraction of the nodal fibres of the S–A node spreading to the auricles by direct continuity of fibres. All the muscle fibres leaving the S–A node can be traced away into the ordinary auricular musculature. There is no trace of any specialised muscle path through the auricular walls. We also found that the spread of contraction from the right to the left auricle took place by two main paths (Figs. 5 and 6). Firstly, fibres from the node passed down to the inter-auricular septum, where fibres from the right crossed the middle line and were continued round the back of the left auricle. Secondly, a broad band of fibres, passing over the tail of the right auricular appendix, swept backwards and passed straight across to the back of the left auricle. Thus we see how the contraction starting in the right auricle causes the contraction of both chambers. The contraction wave, therefore, passes from the sinus node into the auricular muscle of both auricles, and by this means reaches the base of the inter-auricular septum, where it meets another collection of cells termed the auriculo-ventricular node. This is situated on the right surface of the septum just to the right of the opening of the coronary sinus (Fig. 2). The node is very similar to the sinus node, consisting of a network of fine muscle fibres embedded in fibrous tissue. There are many nerve fibres passing into it, and many ganglion cells can be seen amongst the muscle fibres. From this node a bundle of fibres passes across the right side of the inter-ventricular septum towards the *pars membranacea septi*. At this part it divides into two branches; the right one runs down the right side of the septum, the left branch pierces the septum and runs down its left side.

These pass down to the apex and then subdivide into innumerable branches, which are distributed to the muscle fibres of the ventricles, and are so arranged that the muscle fibres of the ventricles contract in a definite order, commencing at the apex and gradually reaching the base.

This sequence of contraction is only possible when the stimulus for contraction reaches the ventricular muscle by means of the bundle. In certain cases the stimulus originates in the ventricular muscle itself on one side or the other, and in this case the spread of contraction does not occur in this sequence, with the result that the power of the contraction suffers.

This different type of contraction evokes a particular kind of electro-cardiogram. If we look at a normal electro-cardiographic tracing (Fig. 7), we find that the waves, or

FIGS. 5 and 6.

*Low-power photomicrographs exhibiting the muscular bands which join the two auricles.*

FIG. 7.
*Normal electrocardiogram.*

FIG. 8.
*Electrocardiogram showing premature beats R'T' arising in the ventricles.*

FIG. 9.
*Electrocardiogram showing auricular fibrillation.*

FIG. 10.

*Polygrams, the upper normal, the lower from a case of auricular failure.*

complexes as they are called, are of three kinds: " P," which is due to the auricular contraction and which occurs from 0.14 to 0.18 of a second before the " R "; " R " denotes the onset of ventricular contraction, and " T " represents the end of ventricular contraction; a normal R and T denotes that the ventricular contraction was called forth by a stimulus reaching the ventricle by the bundle, and is what is called a supra-ventricular complex. An abnormal ventricular contraction arising from the muscle itself gives a very different complex (see Fig. 8).

### Symptom and Signs of Auricular Failure.

In a tracing from a case of auricular failure (Fig. 9), *i.e.* after the onset of auricular fibrillation, it is seen that the ventricular complexes are of the supra-ventricular type, therefore the ventricular contractions are originated by means of stimuli passing down the bundle in the normal way. It is also seen that the " P " wave is absent, but that it is replaced by small irregular waves which occur continuously, being interrupted by irregular spaced ventricular complexes. These correspond with the irregular ventricular beats that are perceptible clinically as the " totally irregular pulse." It was this kind of irregularity that first attracted attention to the syndrome of auricular failure. Mackenzie, Wenckebach and Hering noted the occurrence of a kind of arrhythmia named by them " total " or " perpetual," and associated, as they showed, with the ventricular form of venous curve, *i.e.* one from which the usual auriculo-systolic movements were absent. Lewis carried this further by showing that small irregular waves were found in the electro-cardiogram of total arrhythmia, and that these waves were caused by " auricular fibrillation."

By these successive steps the symptom-complex of auricular fibrillation has become more and more clearly defined, so that now it is easy to recognise.

There are four chief clinical manifestations of auricular failure.

The first cardinal sign is the particular kind of arrhythmia alluded to above. The characteristic fact about this irregularity is that it is complete; the normal rhythm is entirely replaced by a series of beats that vary altogether in force as well as in duration. If the pulse be counted for a number of successive periods of five seconds each, it will usually be found that each period embraces a different number of beats. It is quite different from all other kinds of arrhythmia, not only in that it is more definite, but also in its quality; since in this form of irregularity alone is there no vestige of the original rhythm left. Nearly always it is a quick pulse, less constantly so in the senile than in the rheumatic cases; and the heart rate is quicker than the pulse rate because of a number of small beats which fail to reach the wrist. This discrepancy tends to disappear under successful treatment.

When the substance of this paper was read to the Bristol Medico-Chirurgical Society this type of arrhythmia and its slowing after treatment were well illustrated by Mr. Mitchell's microphone apparatus.

The second feature of auricular failure is the disappearance of all evidence of auricular systole. The auriculo-systolic wave of the jugular pulse is missing, and the venous pulse is of the ventricular type (Fig. 10). That movement in the electro-cardiogram which is the outcome of auricular systole also disappears (Fig. 9). Finally, any presystolic bruit that may be present—as it often is, since it is in a case of late cardiac rheumatism with mitral stenosis that the auricle so frequently fails—also disappears; leaving the diastolic vibrations, if any there be, to end vaguely at an appreciable interval before the next first sound.

Thirdly, all the signs of circulatory stasis are increased.

The patient becomes more breathless, cyanosis is more pronounced and often more livid in character, the jugular veins stand out prominently, the liver becomes swollen and tender, and râles appear at the bases and edges of the lungs.

Finally, in the jugular tracing and in the electrocardiogram (see Figs. 9 and 10) evidence of a rapid irregular movement is visible, occasionally in the former and invariably in the latter.

So far as the patient is concerned, the advent of auricular failure is sometimes so insidious as to attract no attention. Nearly always, however, it brings with it a definite increment of the dyspnœa and other symptoms from which the patient is already suffering. Sometimes the onset is so sudden, and attended by so great an exacerbation of circulatory difficulty, as to cause vomiting and collapse, and to bring the patient down as if with an acute illness. At the other extreme are the persons who have no idea that the action of the heart has undergone any serious change. Sometimes it comes on at first in transient periods of failure, separated from each other by ever shortening intervals. Occasionally, and rather mysteriously, the periodic type of failure may persist for decades, the heart in the intervals appearing to behave quite normally. Pain is a rare concomitant of auricular failure, presumably because it is more especially a symptom of left ventricular insufficiency. Sometimes patients complain of palpitations, but not as often as might be expected from the rapidity of the heart's action and the frequency of the premature ventricular beats that are principal features of the syndrome.

Auricular failure is nearly always a late feature of the chronic forms of cardiac disease. Its incidence in the different types of cardiac disease may be expressed by the following table, extracted from notes on hospital in-patients and on patients recently seen in private practice :—

TABLE I.

| | Hospital Patients. | Private Patients. | Total. |
|---|---|---|---|
| Rheumatic heart disease .. | 48 | 29 | 77 |
| Syphilitic heart disease .. | 2 | 1 | 3 |
| Thyroid heart disease .. | 2 | 4 | 6 |
| Ulcerative endocarditis .. | 2 | 1 | 3 |
| Cardiorenal disease (hyperpiesis) | 3 | 15 | 18 |
| Senile (decrescent) cardio-vascular disease .. .. | 11 | 28 | 39 |
| Various (alcoholism, acute infections, etc.) .. .. | 4 | 13 | 17 |
| | 72 | 91 | 163 |

The analysis of notes from private practice displays a larger percentage of cardio-sclerotics and a smaller one of cardio-rheumatics than the hospital series, but these two remain the dominant types.

This is explained not only by the greater general incidence of those kinds of cardiac disease, but also by the particular predilection which they show for an attack on the auricular functions. The nature of that predilection will appear presently. Indeed, a glance at the nature of the pathological processes of senile heart disease shows that here, at all events, it must be myocardial degeneration that destroys the auricular efficiency. About rheumatic heart disease it is not so obvious; for in its earlier phases there is a definite and highly characteristic form of interstitial myocarditis, and it has been suggested that this, or its residuum, is responsible for upsetting the auricular function. That this is not so is suggested by the following tables, which show that auricular failure is much more often seen in the later decades of cardiac rheumatism than in the earlier phases, when the interstitial inflammation is more pronounced :—

TABLE II.

Of 58 cases of cardiac rheumatism with total arrhythmia

| | | | | |
|---|---|---|---|---|
| 2 | occurred in | patients | aged | 10 or less. |
| 2 | ,, | ,, | ,, | 11–20 |
| 8 | ,, | ,, | ,, | 21–30 |
| 14 | ,, | ,, | ,, | 31–40 |
| 17 | ,, | ,, | ,, | 41–50 |
| 15 | ,, | ,, | ,, | 51 upwards. |

TABLE III.

| Age. | Cases of rheumatic heart disease studied by graphic methods. | Total arrhythmia in. Number. | Per cent. |
|---|---|---|---|
| Up to 10 .. .. | 22 | 1 | 4.5 |
| 11–20 .. .. | 89 | 1 | 1.1 |
| 21–30 .. .. | 45 | 3 | 6.6 |
| 31–40 .. .. | 35 | 5 | 14.2 |
| 41 onwards .. | 25 | 12 | 48.0 |

MORBID ANATOMY.

It was in order to complete a study of rheumatic heart disease, also to satisfy ourselves on the point whether auricular failure is indeed due to degenerative processes, and particularly to find out whether such processes caused failure by a general attack on the auricular muscle or a special attack on the sinu-auricular node, that we undertook an inquiry into the histopathology of auricular failure. To this end a number of hearts from persons dying of cardiac disease with auricular failure was examined. Of these, ten were fully and eight partially examined. The accompanying table shows, among other data, what were the forms of cardiac disease in each case. In addition, a series of twenty-six hearts from persons dying of cardiac disease without auricular failure was also examined fully. The

### TABLE IV.

### CASES IN WHICH THE SIGNS OF AURICULAR FAILURE WERE NOTED DURING LIFE.

#### (a) COMPLETE EXAMINATIONS.

| Ref No. | General Features of Case. | Duration of Failure. | Sinu-Auricular Node. | Auricular Muscle. | Ventricular Muscle. | A-V Connections. | Remarks. |
|---|---|---|---|---|---|---|---|
| 70 | F., 20. Malignant endocarditis supervening on chronic cardiac rheumatism. | A few days. | No abnormality. | Slight cloudy swelling and fatty change. | Slight cloudy swelling and fatty change. | Nil except usual vascular changes of chronic cardiac rheumatism in neighbourhood of central fibrous body. | No active inflammation of myocardium |
| 74 | M., 30. Chronic cardiac rheumatism with mitral stenosis, etc. | Months. | Large hemorrhage into middle of node; about a quarter of its length affected; at the worst point the node was almost cut in two. The blood appeared to come from the neighbouring part of the right auricle. Much of the clot was organised and had been there some time. About one-fifth of the total nodal tissue was destroyed by this hemorrhage. | Marked fatty change. | No abnormality noted. | No abnormality. | |

| Ref. No. | General Features of Case. | Duration of Failure. | Sinu-Auricular Node. | Auricular Muscle. | Ventricular Muscle. | A-V Connections. | Remarks. |
|---|---|---|---|---|---|---|---|
| 75 | F., 55. Chronic cardiac rheumatism with mitral and aortic fibrosis, and recent mitral endocarditis of doubtful character. | Probably of recent onset. | No abnormality. | Advanced fatty change. | Fatty change. | No abnormality. | Old glomerulitis noted in kidney. |
| 82 | M., 50. Chronic cardiac rheumatism, mitral stenosis. | Years. | No abnormality. | Advanced fatty change. | Fatty change only slight in left ventricle. | No abnormality. | — |
| 95 | F., 57. Chronic cardiac rheumatism; decrescent cardiosclerosis; recent endocarditis. | Days only. | Passive hyperæmia. Hypertrophy of A. to node. | Definite fatty change. | Definite fatty change. Cloudy swelling also noted in L.V. wall. Inflammatory foci, especially in I–V septum. | One large lymphocytic focus in early part of bundle. | — |
| 102 | F., 5. Chronic cardiac rheumatism; acute terminal carditis; chronic interstitial nephritis. | Months at least. | Normal; right auricle cavity closely approaches it. | Advanced fatty change in left auricle. Definite fatty change in right auricle. | Definite fatty change in both ventricles. | Normal. | — |

| | | | | | | | |
|---|---|---|---|---|---|---|---|
| 156 | *F., 47. Chronic cardiac rheumatism. Terminal influenza with bronchopneumonia.* | *Weeks.* | *Relative decrease of muscle fibres, which also show some fatty change and hypertrophy. Artery to node shows hypermyotrophy.* Capillaries and arterioles show mild endothelial reaction. | *Great variation in size of fibres. Extremely advanced fatty change.* | *Slight fatty change.* | *Chronic obliterative reaction in artery to node.* | — |
| 107 | F., 34. Chronic cardiac rheumatism with mitral and tricuspid stenosis. | Years. | No abnormality. | Fibres vary much in size. | Slight fatty change. | Arterial hypertrophy. Slight fatty change in muscle fibres. | — |
| 108 | M., 29. Chronic cardiac rheumatism, sudden death. | Months at least. | No abnormality, except doubtful increase of connective tissue at lower pole. | Advanced fatty change. | Fatty change, advanced in places. | Obliterative inflammatory changes in vessels to node. | — |
| 110 | M 17. Acute rheumatic carditis terminating cardiac rheumatism. | About a year ? | Nil, except acute proliferative reaction in artery to node. | Fatty change, advanced in places. | No gross fatty changes. Many inflammatory foci. | Acute obliterative changes in arterioles. | — |
| | | | (*b*) Partial Examinations. | | | | |
| 67 | F., 21. Chronic cardiac rheumatism. Very large heart, mitral fibrosis. | Over a year. | Not examined. | Advanced fatty change. Connective tissue increased. | Definite fatty change. | Not examined. | — |
| 38 | M., 59. Cardiosclerosis with hyperpiesis and terminal streptococcic endocarditis. | Weeks or months. | Not examined. | Advanced fatty change. | No gross changes. | Not examined. | — |

| Ref. No. | General Features of Case. | Duration of Failure. | Sinu-Auricular Node. | Auricular Muscle. | Ventricular Muscle. | A-V Connections. | Remarks. |
|---|---|---|---|---|---|---|---|
| 68 | F., 48. Chronic cardiac rheumatism with mitral stenosis. | 5–10 years. | Not examined. | Advanced fatty change. One or two submiliary nodules. | No gross changes. | Not examined. | — |
| 46 | M., 5. Fulminating rheumatic carditis. | Days. | Partially examined; some inflammatory infiltration. | Saturated with inflammatory reaction. | Widespread and intense inflammatory reactions. | Partially examined. Some inflammatory reaction. | — |
| 100 | F., 31. Chronic cardiac rheumatism, with mitral stenosis; terminal endocarditis. | More than a year. | Not examined. | Cloudy swelling and segmentation. | Cloudy swelling and segmentation. | Not examined. | — |
| 69 | F., 41. Chronic cardiac rheumatism with mitral stenosis. | More than a year. | Not examined. | Advanced fatty change. | Definite fatty change. | Not examined. | — |
| 57 | M., 50. Alcoholic degeneration of myocardium. | Months. | Partially examined. No change. | Advanced fatty change. | Definite fatty change. | Not examined. | — |
| 64 | Child dying of diphtheria. | Days. | Not examined. | Advanced fatty change and cloudy swelling. | Definite fatty change and cloudy swelling. | Not examined. | — |
| 66 | M., 48. Chronic cardiac rheumatism, mitral stenosis, etc. | Years. | Not examined. | Advanced fatty change, etc. | Slight or definite fatty change. | Not examined. | — |

difference between partial and complete examination is that in the latter serial sections were cut of the sinu-auricular node and the auriculo-ventricular connections, whereas in the partial examinations these series were omitted.

In only one case of auricular failure that was fully investigated was there any lesion of the sinu-auricular node that could conceivably have put an end to its effective action; and of that case, even, it is as true as it is of the others that significant degenerative changes were scattered freely throughout the auricular walls. Further, it is not unreasonable to think that the hemorrhage that had destroyed a part of the sinu-auricular node was a consequence, and not a cause, of auricular failure. Apart from this one instance the node was entirely free from gross morbid change.

On the other hand, in every case of auricular failure the musculature of the auricular walls had reached a pitch of deterioration so extreme that it was demonstrable by routine histological methods. The fatty change that has been accepted as the readiest index of that deterioration was most advanced in the parts of the auricular muscle that lay just under the endocardium. In some hearts the areas lying near the tip of the appendices displayed this most clearly, in others the change was at its maximum in the main auricular walls. Shrinkage of the decadent muscle cells brings into unnatural prominence the delicate interstitial stroma of the auricular walls. In the walls of the ventricles, on the other hand, changes such as these are barely discernible, and only in small and scattered patches.

This contrast between the decadence of the auricles and the integrity of the ventricular walls is brought into greater relief if it is compared with two other sets of facts; first, with the absence of any such marked contrast from the

26 "control" specimens[1] alluded to above, and second, with the freedom of the auricular walls from inflammatory lesions in cases of acute cardiac infection. To each of these generalisations there are exceptions. For example, records 58, 61 and 76 were of cases of chronic cardiac rheumatism, with predominating mitral lesions but no signs of auricular fibrillation in life. In these the auricular muscle was grossly degenerate, much more so than that of the ventricles, showing that in such cases this contrast becomes apparent even before the arrhythmia that is characteristic of auricular failure sets in. Again, record No. 46 is that of a case of acute rheumatic carditis in which the inflammatory lesions of the auricular muscle surpassed those of the ventricles both in diffuseness and in intensity. Yet both of these exceptions do but support those generalisations which we feel justified in drawing from our observations of the histological changes that accompany auricular failure; first, that it is the whole auricular wall that "fails" and not the sinu-auricular node; and, second, that failure is usually the outcome of deterioration of the muscle cell itself. How profound that deterioration may be is well seen in the accompanying photomicrographs (Figs. 11 and 12).

### Prognosis and Treatment.

Auricular failure, being practically always associated with advanced organic change, brings into any case of heart disease an element of gravity; first, because it is itself strong evidence of serious myocardial deterioration; second, because its rapid irregular rhythm lays on the ventricle a new burden. It is on this second fact that the prognosis of auricular failure largely turns. If the ventricles are already

[1] It is true that the texture of the auricular wall is such that it is easier to detect these changes in it than in the denser ventricular muscle, but this difference by no means explains away the whole of the contrast to which we refer.

FIGS. 11 and 12.

*Photomicrographs showing myocardial degeneration in the auricular wall in auricular failure. The upper is a low-power and the lower a high-power photograph.*

*(Blocks from " Rheumatic Heart Disease," kindly lent by Messrs. John Wright and Sons Ltd.)*

diseased and ineffective when the auricular functions fail, it is unlikely that treatment will afford a large measure of relief. Some relief there will be if, under appropriate treatment, the auricles are induced to beat regularly, or, failing this, fewer stimuli are allowed to pass through into the ventricles ; and if the ventricles are still in fair condition, the control of auricular failure furnishes for therapeutics one of the most striking opportunities of triumph in the whole field of medicine.

We have many examples of persons whose cardiac symptoms were so far allayed and kept in check by these means, that they were enabled to carry on their business for many years in comparative comfort. But others are insusceptible of relief ; and this is partly because, in a few instances, the drugs of which we are wont to expect a good deal prove ineffective, but more often because the ventricles are so far gone in disease that even when they have been relieved of the burden thrown upon them by failure of the auricular functions they cannot do their work effectively.

Many other elements may be introduced into a discussion of the prognostic significance of auricular failure ; but we wish to limit ourselves here to a statement of our belief that there are three principal sets of facts to be considered if we are to forecast truly the expectations of a case of cardiac disease in which the syndrome of auricular fibrillation is discovered. Those three are, first, the nature of the underlying lesion, whether it is transient or progressive, and, second, the state of the ventricles as far as this can be ascertained ; third, the behaviour of the fibrillating auricle under the influence of the appropriate drugs.

In practically all cases of cardiac disease presenting signs of auricular failure the first indication is to reduce the cardiac speed and thus to lengthen diastole. This indication is met by the use of digitalis. For patients whose symptoms are only moderately severe moderate doses will suffice. For example, people who are not so ill but that they can attend an out-patient department take 15 minims of the tincture, or a pill containing one or two grains of the powdered leaf, every four hours. But it is for those who, when they first come under treatment, are urgently and even dangerously ill, that we would specially urge the use of digitalis in massive doses. Two or three years ago Eggleston of New York began to give the drug in a new way for cases of this kind. He showed that it was safe to give the dose which was just below toxicity in a single mass. The equation relating that dose to the patient's body weight is such that for an adult of average weight the dose would be three or four drachms of the tincture.

We have secured really remarkable results from a modification of this method, followed for two or three years; results paralleled in the experience of others who, independently of ourselves, adopted closely similar modifications of the Eggleston method. Our plan is to get three drachms taken in the first twenty-four hours. The accompanying chart (Fig. 13) shows how this plan brought down the heart rate in one case of this kind. We have seen lives prolonged for a year or more in at least a dozen instances, that would indubitably have been lost under any less thorough treatment. It is wonderful to see the relief afforded to the patient; and this, with reduction of the heart and pulse rates, and increase in the output of urine, may be confidently looked for in a majority of the rheumatic cases. We split our dose up in various ways; a good method is to give an initial dose of one drachm of the tincture, and to follow this by half-drachm doses at intervals of four hours till three drachms in all have been taken. The drug is stopped for twenty-four hours or more, and then resumed at a rate of fifteen minims every four hours. This kind of plan has been followed in somewhere about fifty cases, and we have never yet seen toxic symptoms. More than once we have seen vomiting actually checked by digitalis in massive doses. We wish to lay the greatest possible emphasis on this, that the syndrome of auricular failure constitutes an absolute indication for the use of digitalis; that no other drug will take its place, and that no other syndrome is anything like so sure of relief from its use.

FIG. 13.

*Chart illustrating response of heart rate and pulse rate to treatment by massive doses of digitalis.*

(*Block from "Rheumatic Heart Disease," kindly lent by Messrs. John Wright and Sons Ltd.*)

The other drug that may achieve striking success in this syndrome, quinidine, has an action totally different from that of digitalis. Whereas digitalis reduces the cardiac speed without pretending to restore the normal rhythm, quinidine aims at an actual restoration of the normal rhythm. Our own experience of quinidine, based on more than thirty cases, shows that in about one-third it has no effect at all; in another third it has a fleeting effect on the rhythm; in another third it achieves a more lasting success. But there are several reservations to be added to this statement. In the first place, most of the successes are not really permanent; they only last a few days or weeks, and then the abnormal rhythm returns. Second, in a few instances a fibrillation rhythm turns over to normal either spontaneously or under the influence of digitalis, and it is possible that some of the quinidine successes would have proved amenable to other plans of treatment. Then, again, the use of quinidine is not without risk. In one instance we have known sudden death occur during its administration. Other cases of the same kind are on record, as well as a number in which revival of auricular systole by quinidine was followed by embolism from the disturbance of clot which had settled down in the paralysed auricular appendix. This drug should therefore be used only for the most carefully selected cases, and under close supervision, the patient being kept in bed meanwhile. If it is decided to give it, the plan followed by one of us (C. F. C.) is to administer a single dose of 5 grains of the sulphate, to see if the patient has an idiosyncrasy for the drug. If not, it is given in 5 grain doses, in pill or cachet, every four hours till the normal rhythm is restored. It is worth going on for four days to a week before abandoning the attempt as fruitless, but as a rule if quinidine is going to cure it does so at once. The reversion from the abnormal to the normal rhythm takes place abruptly and without any half-way stages. To maintain the normal rhythm it is well to give quinidine, 5 or 10 grains daily, for some long time.

The plan followed by the other of us (C. E. K. H.) differs in details only from the above. A single dose of 0·4 gramme (6 grains) is given on the first day, and if this is followed by no untoward results a similar dose is given every two hours, for five doses per day, this being continued for five days if the normal rhythm is not recalled in a shorter time.

### Physiological Considerations.

These therapeutic procedures gain much in interest as well as in reliability because they are founded on a scientific basis, an application of pharmacology to the relief of diseased functions. As the result of work done by Lewis and his co-workers in London we now know the mechanism of the conditions known as auricular flutter and fibrillation. If a complete circle of muscle be stimulated to contract by an induction shock at a certain spot a wave of contraction starting from that point will pass round each side of the ring until it "meets itself" at the reverse side of the ring, when the contraction ceases. If, however, this ring of muscle has been made to contract by rapidly repeated induction shocks just before the experiment, it is found that during the partial recovery of refractoriness the wave of contraction may be stopped from passing up one side of the ring while it passes up the other side. This wave will then pass completely round the ring, and when it reaches its starting-point it will, provided the muscle has sufficiently recovered from its last contraction, start again round the ring, and so on indefinitely. The only condition necessary for the continuance of this phenomenon is that the time of partial recovery from the refractory period must be a little less than the time taken for the contraction to pass round the ring of muscle; in other words, there must be a gap between the muscle that is in an absolutely refractory state and the advancing wave of contraction. The time taken to complete the circuit depends chiefly upon the length of the path round the ring; the rate of the contraction wave and the length of the refractory period being more or less constant. Lewis has found that a similar condition of "circus movement" occurs in experimentally induced flutter and fibrillation in dogs, and there is no doubt that the conditions in man are caused by the same mechanism.

The difference between fibrillation and flutter is mostly in the length of the path. In flutter the path is longer and the gap between the advancing wave of contraction and the refractory path is wide, and there is no hindrance to the path of the wave round the ring, so each circuit takes the same time, *i.e.* 1/300 of a second; therefore, in auricular flutter the auricle contracts 300 times a minute, the wave of contraction passing from the ring of muscle out to the rest of the auricular fibres. In fibrillation the path is shorter and the gap is narrower; consequently the muscle is more disinclined to contract than in flutter; in other words, the partial refractoriness is nearer the absolute. The wave of contraction, therefore, finds difficulty in passing round the ring of muscle, and has to discover a path for itself among fibres of which some are impassable, while others can be passed only with difficulty. No two circuits are composed of the same fibres, and the time taken to complete the circuit is not constant. For this reason the auricular rhythm in fibrillation is irregular, and since it takes about 1/450 of a second to complete the circuit, there are as many as 450 circuits of the ring of muscle per minute. The spread of contraction into the auricular muscle is irregular and much more rapid, some of the muscle is refractory, and there is no co-ordinate conduction from bundle to bundle; hence there is no co-ordinate contraction of the auricles, but the muscle is continuously quivering from the irregular contractions of individual bundles of fibres, giving the muscle the appearance of a fibrillary tremor like that met with in progressive muscular atrophy. Lewis has shown that in flutter the ring of muscle which is acting in this way is the muscle surrounding the apertures of the two venæ cavæ, while in fibrillation the wave of contraction circles round one of the veins. In auricular flutter the ventricle does not respond to every auricular beat, as heart block of varying grade may be present, thus acting as a protecting mechanism ; for the heart would rapidly fail if the ventricles tried to contract at a rate of 300 per minute. Similarly in fibrillation the auriculo-ventricular connections refuse transmission to a majority of the stimuli coming down from the auricles. It is in a strengthening of this inhibitive action of the auriculo-ventricular connections that the therapeutic value of digitalis lies.

Digitalis used in fairly large doses has a depressant action on the junctional tissues between the auricle and ventricle. It delays or decreases conductivity of the bundle, thereby preventing a large number of the stimuli from the fibrillating auricle reaching the ventricle. Hence the ventricle is slowed.

Its action on the fibrillating auricle is, as a rule, to quicken the rate of the contraction wave and to shorten the refractory period, consequently it does not alter the auricular rhythm, nor does it tend to stop the fibrillation. So that although it markedly improves the condition of the patient, one must not expect it to cure the fibrillation.

Quinidine acts in the opposite way ; it tends to increase the ventricular rate, but on the auricles it has two actions, (1) it slows the rate of contraction, (2) it lengthens the refractory period. Sometimes one predominates, at others the other is the more marked. If its chief action is to lengthen the refractory period, the advancing wave of contraction round the ring of muscle catches up the refractory muscle, the gap is obliterated, the circus movement comes to an end and normal rhythm recommences. If the slowing of the rate of contraction predominates the time taken to complete the circuit is lengthened, the gap is unaltered and the fibrillation continues. This is the reason why quinidine sometimes stops fibrillation, and at others appears to have no such action.

## Figures and Tables

**Fig. 1. f1:**
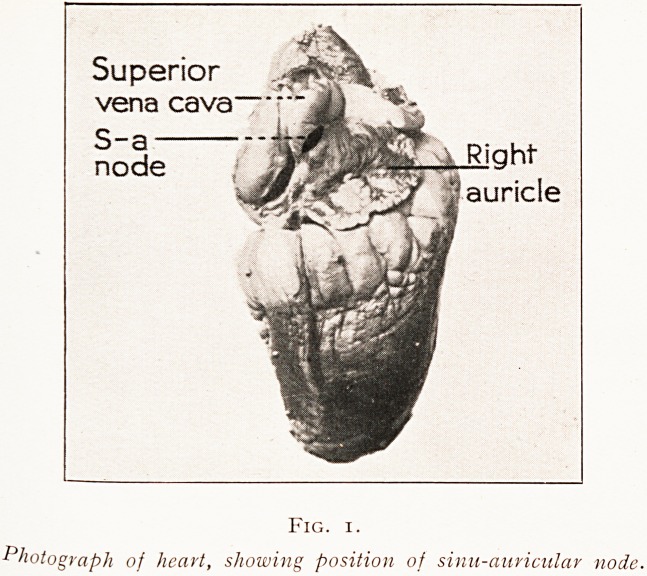


**Fig. 2. f2:**
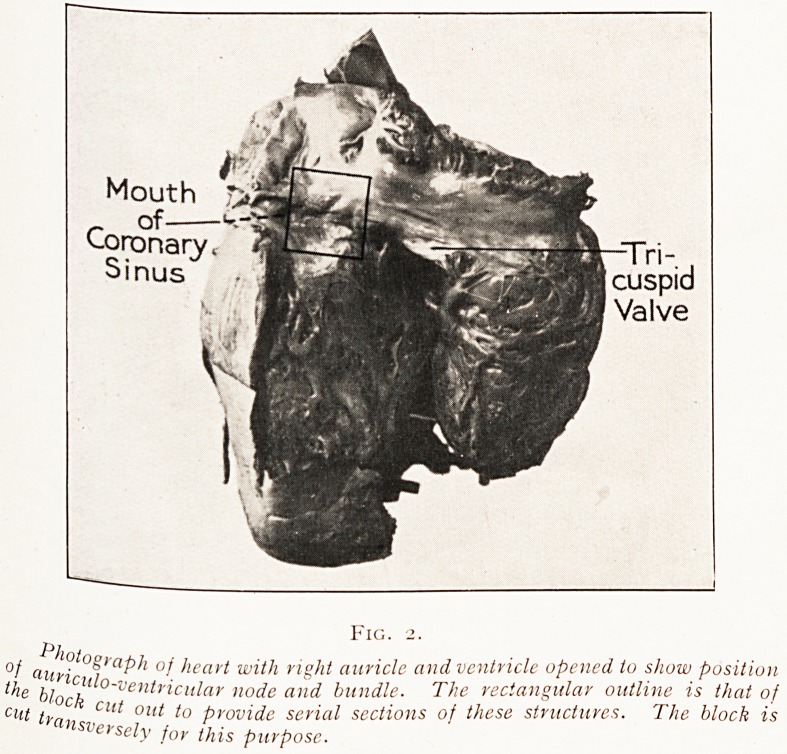


**Figs. 3 and 4. f3:**
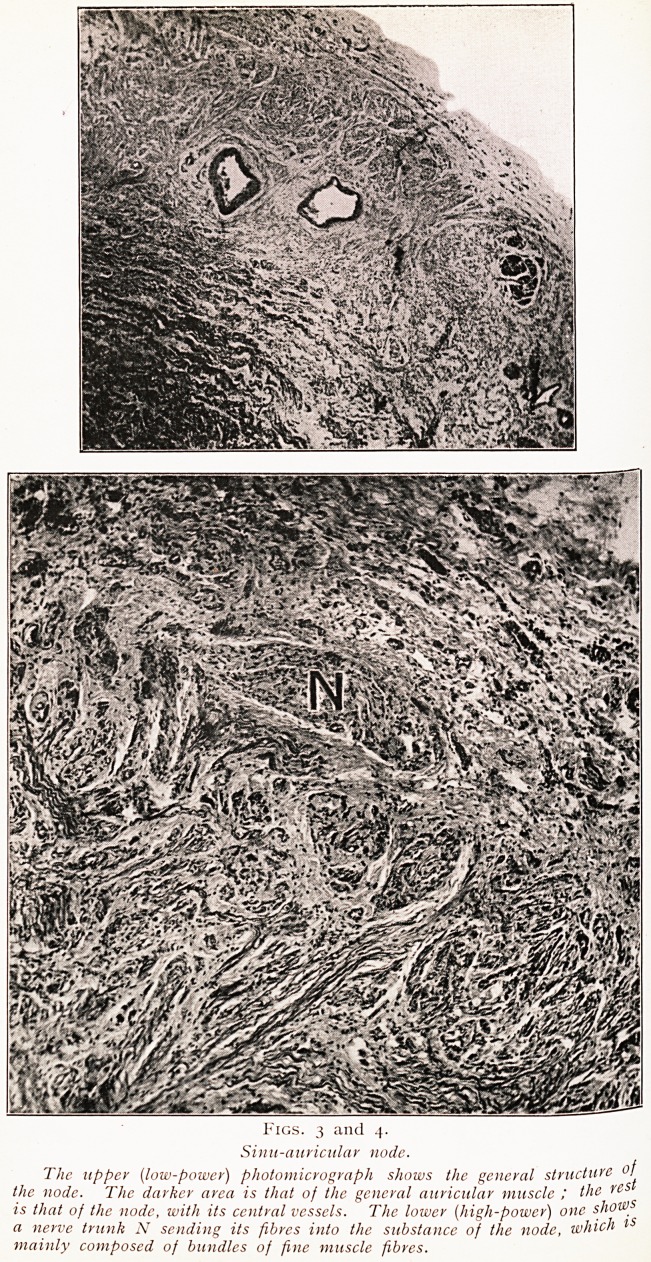


**Figs. 5 and 6. f4:**
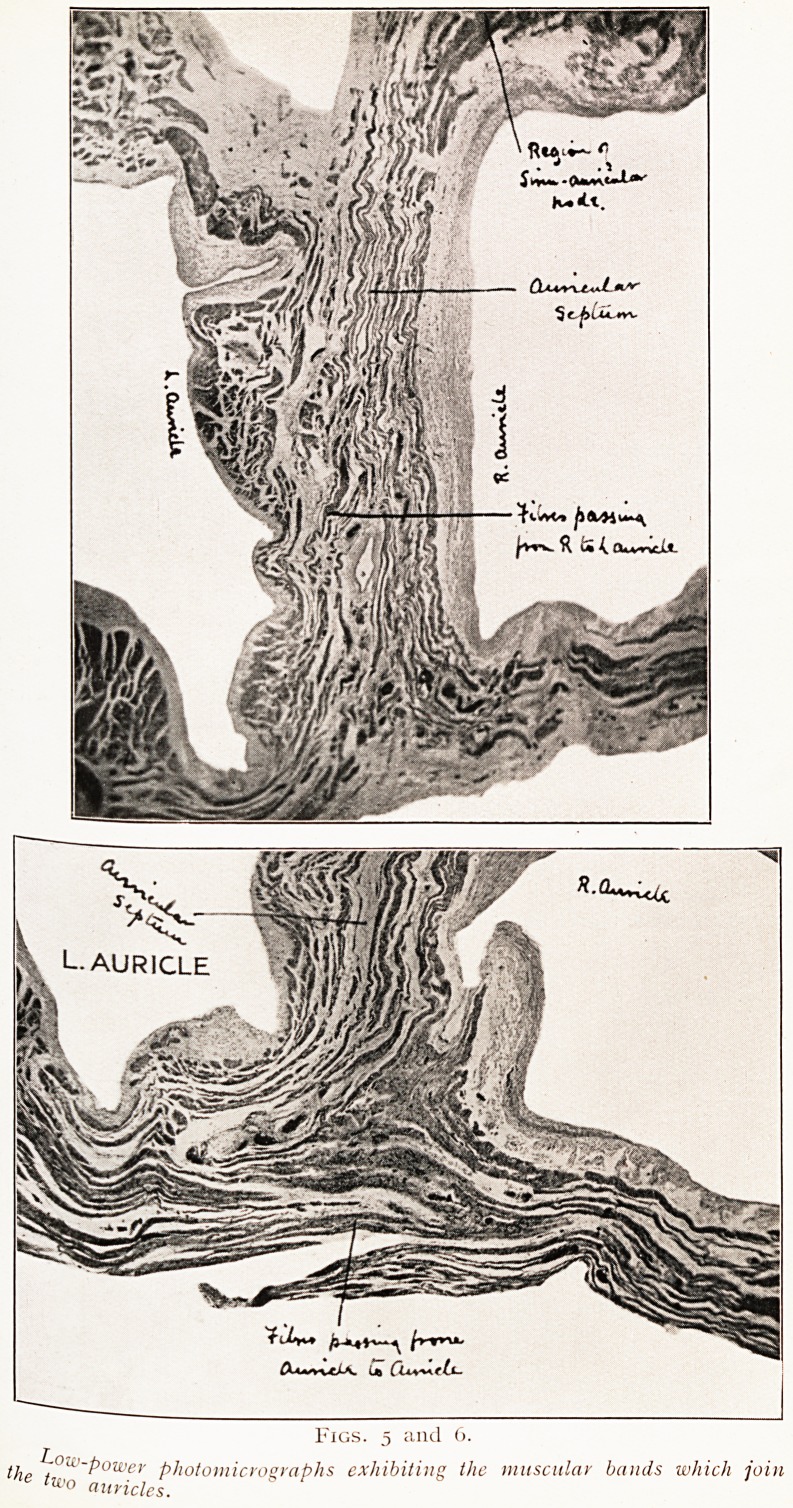


**Fig. 7. f5:**
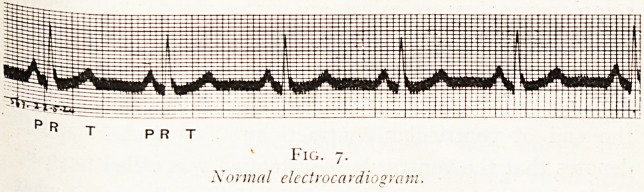


**Fig. 8. f6:**
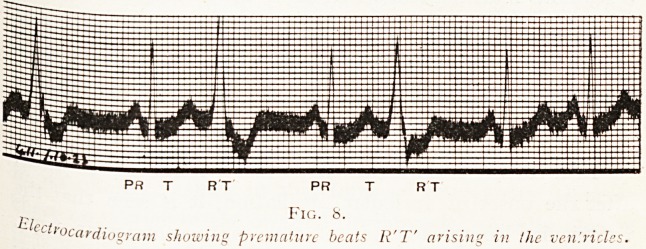


**Fig. 9. f7:**
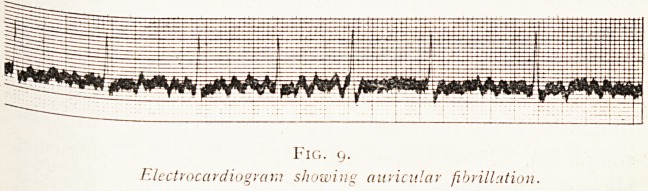


**Fig. 10. f8:**
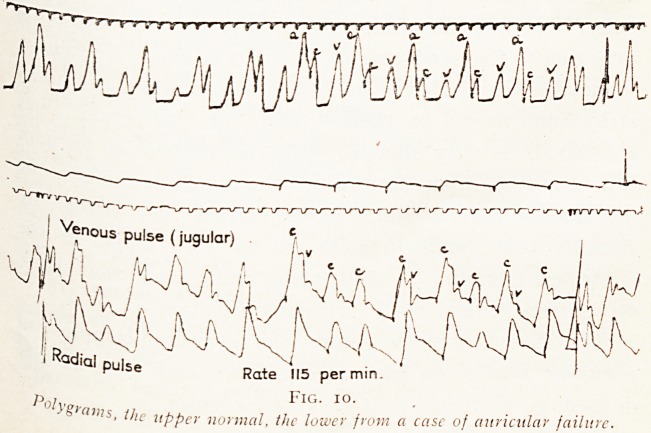


**Figs. 11 and 12. f9:**
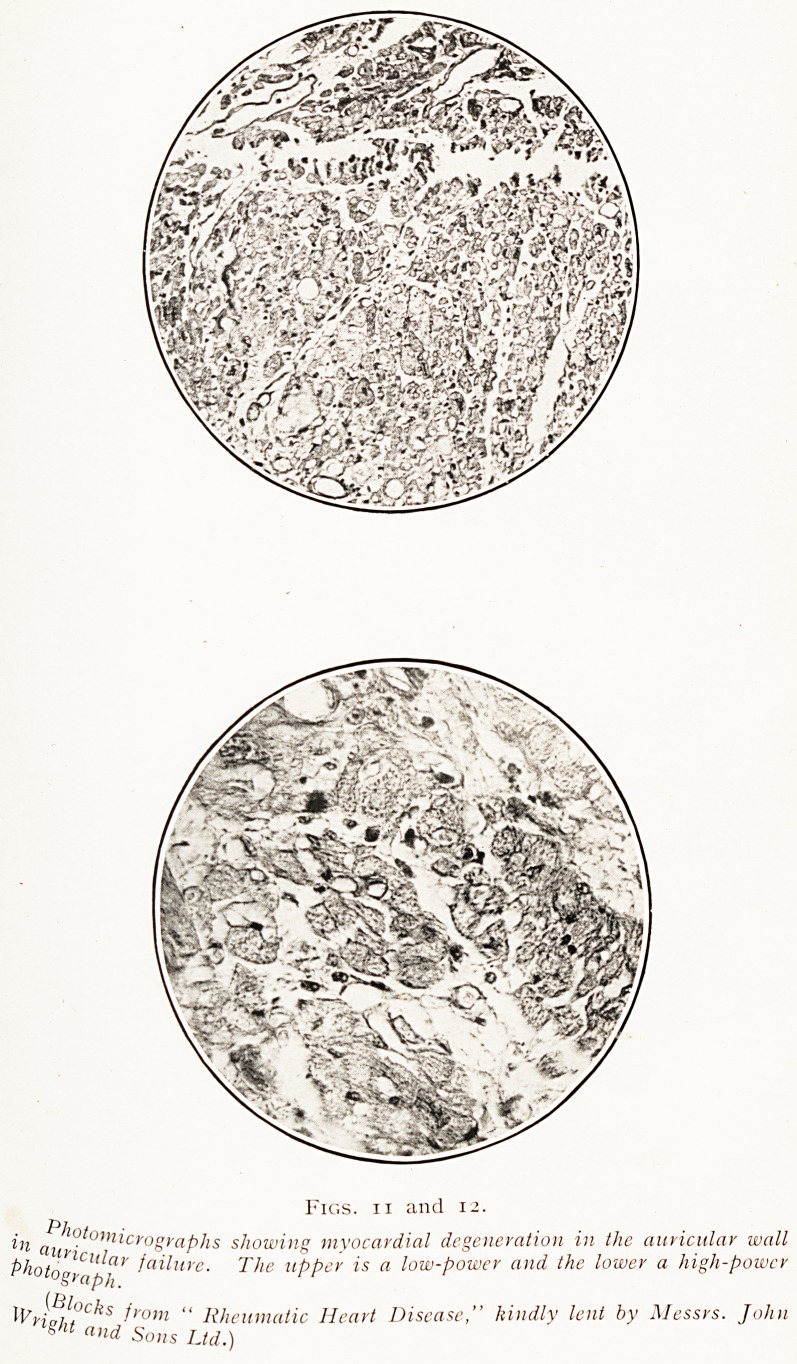


**Fig. 13. f10:**